# TRIM25 in the Regulation of the Antiviral Innate Immunity

**DOI:** 10.3389/fimmu.2017.01187

**Published:** 2017-09-22

**Authors:** María Martín-Vicente, Luz M. Medrano, Salvador Resino, Adolfo García-Sastre, Isidoro Martínez

**Affiliations:** ^1^Unidad de Infección Viral e Inmunidad, Centro Nacional de Microbiología, Instituto de Salud Carlos III, Madrid, Spain; ^2^Department of Microbiology, Icahn School of Medicine at Mount Sinai, New York, NY, United States; ^3^Global Health and Emerging Pathogens Institute, Icahn School of Medicine at Mount Sinai, New York, NY, United States; ^4^Department of Medicine, Division of Infectious Diseases, Icahn School of Medicine at Mount Sinai, New York, NY, United States

**Keywords:** TRIM25, innate immunity, ubiquitination, virus, E3 ubiquitin ligase

## Abstract

TRIM25 is an E3 ubiquitin ligase enzyme that is involved in various cellular processes, including regulation of the innate immune response against viruses. TRIM25-mediated ubiquitination of the cytosolic pattern recognition receptor RIG-I is an essential step for initiation of the intracellular antiviral response and has been thoroughly documented. In recent years, however, additional roles of TRIM25 in early innate immunity are emerging, including negative regulation of RIG-I, activation of the melanoma differentiation-associated protein 5–mitochondrial antiviral signaling protein–TRAF6 antiviral axis and modulation of p53 levels and activity. In addition, the ability of TRIM25 to bind RNA may uncover new mechanisms by which this molecule regulates intracellular signaling and/or RNA virus replication.

## Introduction

**Figure 1:**
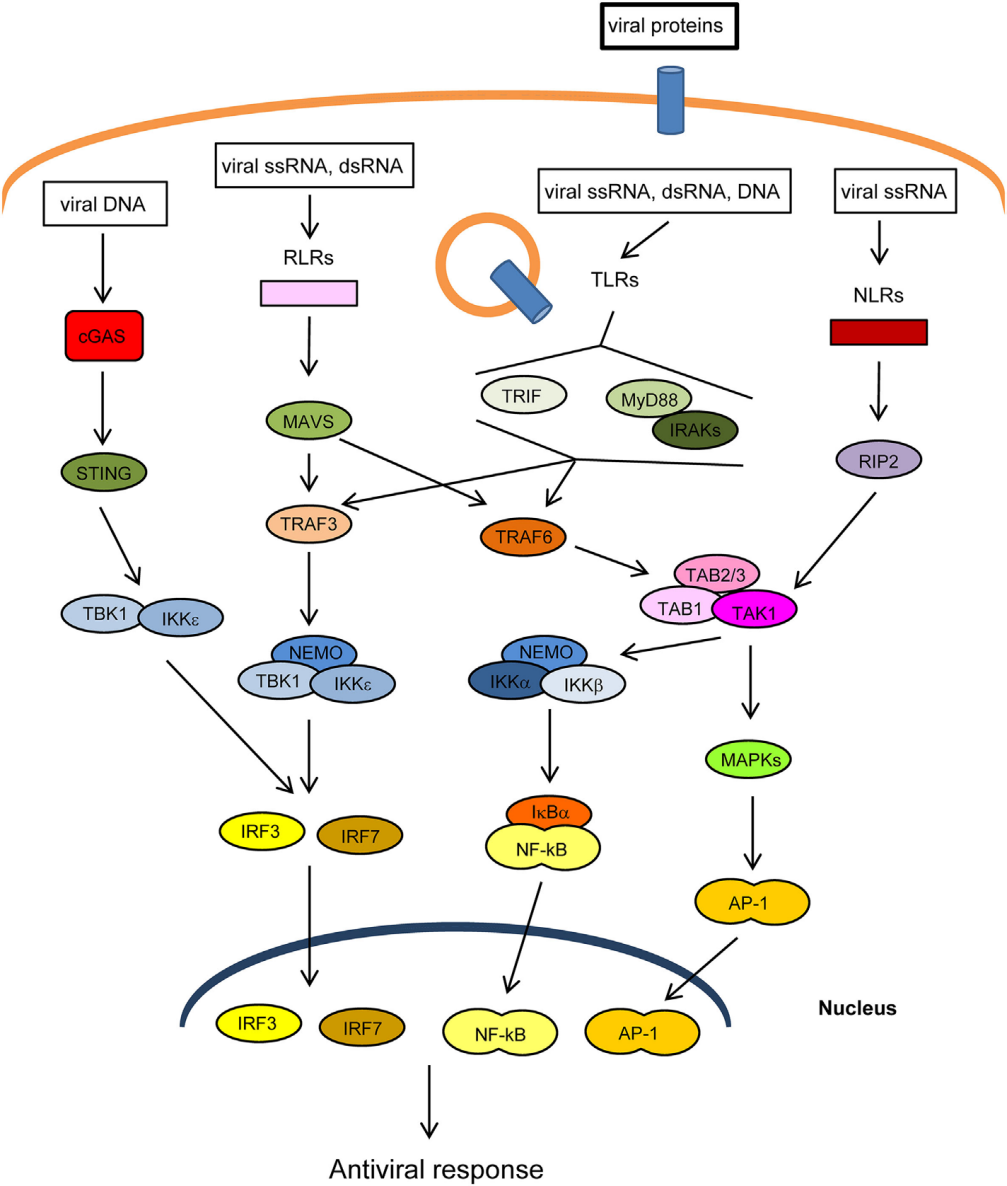
Main pathways of antiviral innate immunity. After recognition of pathogen-associated molecular patterns (PAMPs) by pattern recognition receptors (PRRs) [cGAS, RIG-I-like receptors (RLRs), toll-like receptors (TLRs), NOD-like receptors (NLRs)], the signal is propagated through adaptor proteins (STING, MAVS, MyD88, TRIF) to members of the TRAF ubiquitin E3 ligase family (TRAF3/6) and to kinase complexes (IKKα/β/γ, TBK1/IKKε, TAK1/TAB 1/2/3, MAPKs), which trigger the activation of IRF3/7, NF-κB and AP-1 transcription factors and the expression of IFN-I, proinflammatory cytokines, and other antiviral genes. The activity or stability of most of these proteins is regulated by ubiquitination/deubiquitination processes.

The innate immune response is the first line of defense against invading pathogens. At the cellular level, this response is stimulated by several cellular “pattern recognition receptors” (PRRs) that recognize microbial-specific molecules termed “pathogen-associated molecular patterns” (PAMPs). Bacterial PAMPs include lipopolysaccharide (LPS), flagellin, peptidoglycan, and cyclic dinucleotides, among others. Although viral proteins are able to stimulate specific PRRs, the main viral PAMPs are nucleic acids, including double-stranded RNA (dsRNA), uncapped single-stranded RNA, and viral DNA (1–3). Two main groups of PRRs that recognize virus-derived nucleic acids have been described according to their location: (1) membrane-spanning toll-like receptors (TLRs), which detect viral RNA or DNA in endosomes and (2) cytoplasmic sensors, including RIG-I-like receptors (RLRs), NOD-like receptors (NLRs), which recognize cytoplasmic viral RNA, and a group of structurally unrelated intracellular viral DNA sensors (1) (Figure 1). Binding of PAMPs to PRRs leads to the activation of intracellular signaling pathways that produce type I interferons and inflammatory cytokines (4). These pathways converge at the level of several kinases of the inhibitor of nuclear factor kappa-B [Ikβ] kinase (IKK) family: the canonical complex composed of IKKα, IKKβ and the regulatory subunit IKKγ/NEMO, and the non-canonical IKKε and TANK-binding kinase-1 (TBK1) (Figure 1). The IKKα/β/γcomplex activates nuclear factor kappa B (NF-κB), while TBK1/IKKε activates IFN-regulatory factors 3 and 7 (IRF3/7) (5, 6). In addition, PRRs trigger phosphorylation of several mitogen-activated protein kinases (MAPK), which leads to activation of activator protein-1 (AP-1). Upon translocation into the nucleus, the transcription factors NF-κB, IRF3/7, and AP-1 induce the expression of IFNs and other cytokines and antiviral molecules (7) (Figure 1). These molecules not only inhibit viral replication, assembly, and spread but also play a crucial role in activating the adaptive immune response (8).

The innate immune response is crucial for limiting viral infections, but it has to be tightly regulated to avoid immune-mediated tissue damage, excessive inflammation, and auto-immunity (9–11). In this regard, the complex interactions between molecules of intracellular signaling networks leading to the antiviral response are regulated by various mechanisms, of which post-translational protein modification is one of the most relevant. It has been known for a long time that phosphorylation plays a crucial role in the regulation of such networks but, in recent years, reversible conjugation to ubiquitin and ubiquitin-like proteins has emerged as an additional and central mechanism regulating intracellular signaling (12–16).

## Ubiquitination/Deubiquitination Processes Adjust the Innate Immune Response

**Figure 2:**
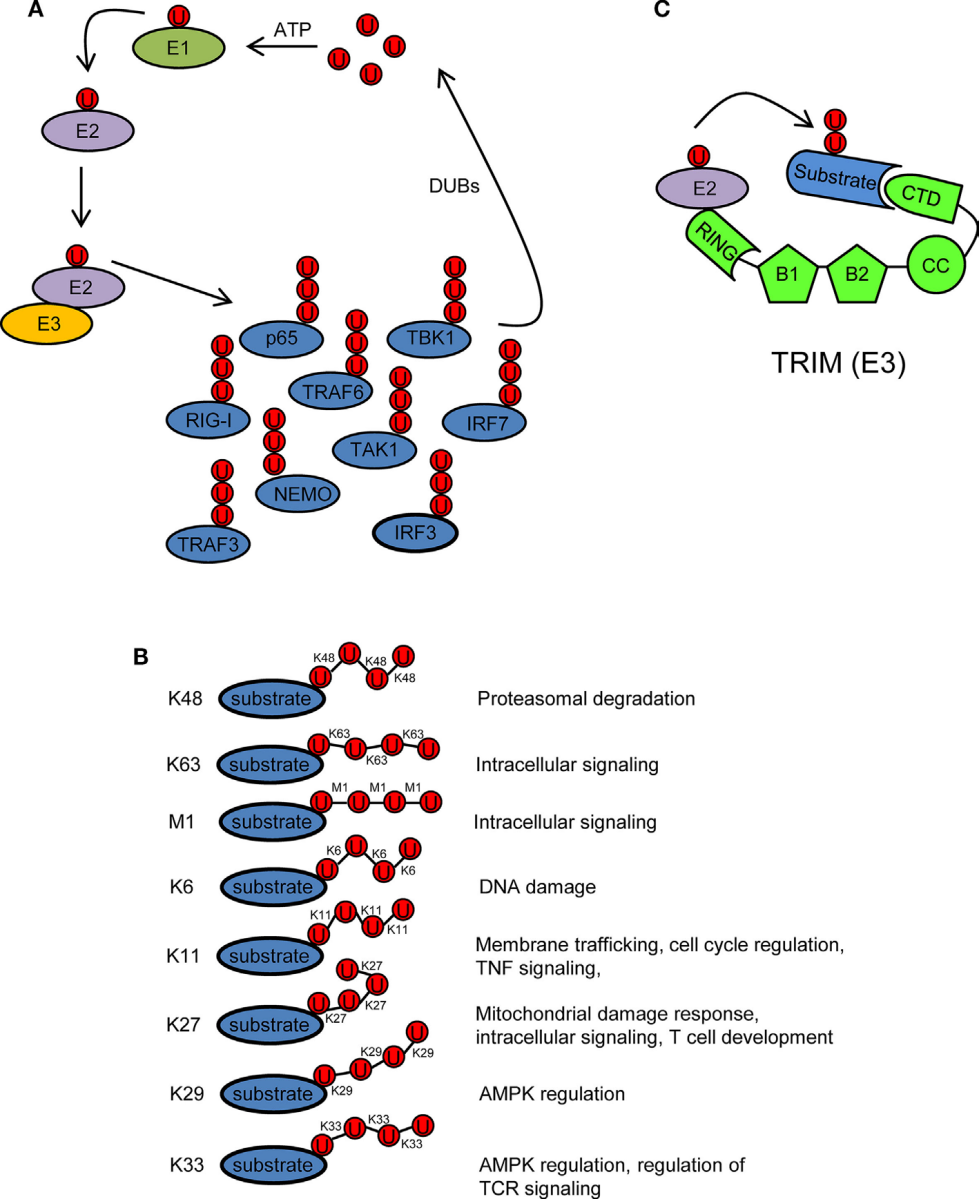
Ubiquitination/deubiquitination regulates intracellular immune pathways. **(A)** Ubiquitination is a three-step enzymatic process by which ubiquitin is covalently attached to target proteins, including ubiquitin itself. This process is reversed by deubiquitinases (DUBs). The activity of several molecules (p65, RIG-I, TRAF3/6, NEMO, IRF3/7, etc.) participating in intracellular immune signaling pathways is regulated by ubiquitination/deubiquitination modifications. E1, ubiquitin activating enzyme; E2, ubiquitin conjugating enzyme; E3, ubiquitin ligase. **(B)** Ubiquitin linkage types and their functional roles. Different ubiquitin linkages led to different conformations of ubiquitin chains that are recognized by proteins containing ubiquitin-binding domains. **(C)** Schematic representation of a tripartite motif (TRIM) molecule, including the conserved N-terminal TRIM [really interesting new gene (RING), B box, and coiled-coil (CC) domains] and a C-terminal variable domain (CTD). Ubiquitin-loaded E2 and substrate are recognized by the RING and CTD domains of TRIM, respectively. This brings both molecules in close proximity and facilitates substrate ubiquitination. Ubiquitin molecules in **(A–C)** are represented by a U inside a red circle. The number of Us does not represent the actual level of ubiquitination.

Ubiquitin is a 76 amino acid (8.5 kDa) protein that can be covalently ligated to lysine residues of a target protein through its conserved C-terminal di-glycine motif (17). Ubiquitination is a three-step enzymatic process involving three enzymes with distinct functions: E1 activating, E2 conjugating, and E3 ligating (Figure 2A). The first step is the formation of a thioester linkage between ubiquitin and the E1. In the next step, ubiquitin is transferred from E1 to the active-site cysteine of E2. Finally, E3 assists the formation of an isopeptide bond between the C-terminal glycine of ubiquitin and a lysine residue of a target protein (18–21). Ubiquitin itself can be ubiquitinated and form polyubiquitin chains (polyubiquitination). Ubiquitin contains seven lysine residues (K6, K11, K27, K29, K33, K48, and K63) on which diverse chain types of polyubiquitin can be assembled (20) (Figure 2B). In addition, ubiquitin chains can also be linked in a linear (M1) fashion by attachment of the C-terminal glycine of an ubiquitin to the N-terminal methionine of another ubiquitin, resulting in a head-to-tail polyubiquitination (22) (Figure 2B). Different ubiquitin linkages fulfill different functions (Figure 2B). K48- and K63-linked polyubiquitin chains are the best characterized. K48 linkage targets proteins for proteasomal degradation, whereas K63 and M1 linkages regulate intracellular immune signals (14–16, 23, 24). The other linkage types are referred as “atypical” and have not been studied in much detail (25). Each chain type has a different three-dimensional conformation that is recognized specifically by ubiquitin-binding proteins (UBPs). This recognition is essential for the transmission of intracellular signaling (26–28). Recently, it has been reported that in addition to the above described covalently attached polyubiquitin chains, unanchored polyubiquitin chains also contribute to the activation of intracellular pathways leading to the onset of the antiviral response (29, 30).

Ubiquitination is reversed by deubiquitinases (DUBs) that detach ubiquitin from the substrate (Figure 2A). This usually leads to termination of immune signaling. In this way, ubiquitination/deubiquitination processes dynamically regulate the early innate immune response and prevent immune-mediated host damage (13).

## Tripartite Motif (TRIM) Proteins are E3 Ubiquitin Ligases

Tripartite motif proteins have E3 ubiquitin ligase activity and form a large family with over 70 members in humans. Their name is derived from the fact that they share three conserved N-terminal domains: a really interesting new gene (RING) domain, one or two B-Boxes (B1/B2) and a coiled-coil (CC) domain. By contrast, the C-terminal region is of variable composition (31, 32) (Figure 2C). The RING and B-box domains are both cysteine–histidine-rich domains that bind zinc atoms. The RING domain recognizes the ubiquitin-loaded E2 conjugating enzyme and promotes ubiquitin conjugation to target proteins (32–35) (Figure 2C). Based on conserved structural features with the RING domain, it has been suggested that the B-boxes could also contribute to E3 ubiquitin ligase activity of TRIM proteins (36, 37). In some TRIMs, B-box domains mediate self-association, which may be important for TRIM oligomerization (38–40). The CC domain is necessary for dimerization of TRIM ligases (41). Finally, the variable C-terminal domain may mediate interaction with specific substrates (Figure 2C). The most common C-terminal TRIM domain is the PRY-SPRY or B30.2 domain, which has been proposed to be involved in protein–protein interactions and/or RNA binding (42).

Tripartite motif E3 ligases regulate many cellular processes, including development, cell growth, differentiation, cancer, and innate immune response (32, 43–45). Several TRIMs have been reported to exhibit antiviral activity either directly or through regulation of antiviral cell signaling (45–53).

## TRIM25 Regulates Intracellular Signaling

**Figure 3:**
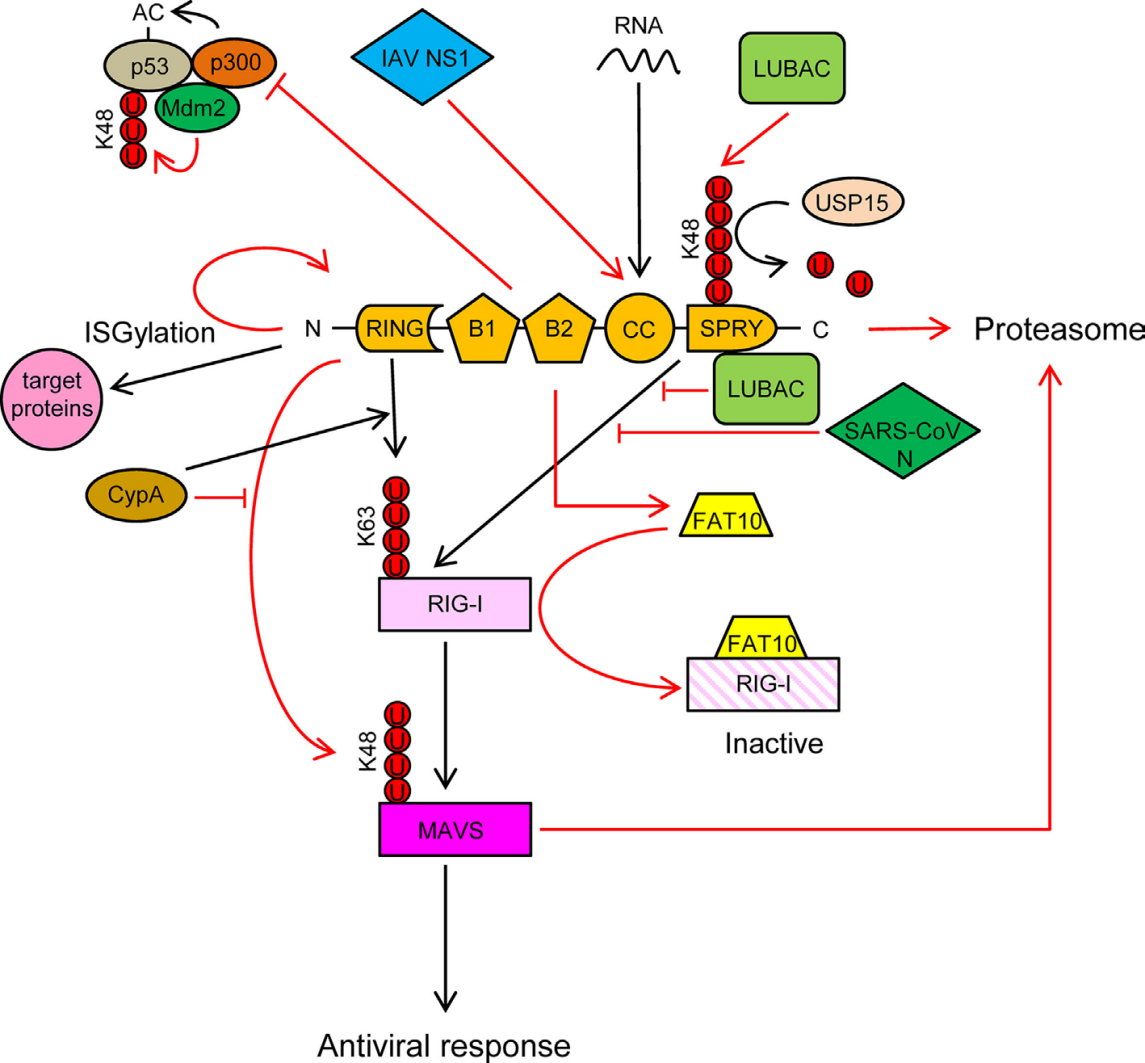
TRIM25 participates in several innate immune-related processes and its activity is regulated by cellular and viral proteins. See text for detailed description of the individual processes. Ubiquitin molecules are represented by a U inside a red circle. Interactions and inhibitory processes are represented by red lines. The number of Us does not represent the actual level of ubiquitination.

TRIM25 is a type I and type II IFN-inducible E3 ligase (54) that was first identified as an “estrogen-responsive finger protein” (EFP) (55). It is composed of a RING domain, two B-boxes domains, a CC dimerization domain and a C-terminal SPRY domain (Figure 3).

TRIM25 is involved in numerous cellular processes, such as development, cancer, and innate immunity (56). Regulation of RIG-I signaling by K63-linked polyubiquitination is one of the best-characterized roles of TRIM25 (57). Recognition of viral RNA by RIG-I exposes its 2CARD domain for binding to the C-terminal SPRY domain of TRIM25 (Figure 3). The RING E3 ligase activity of TRIM25 then conjugates K63-polyubiquitin chains to residues K99, K169, K172, K181, K190, and K193 of RIG-I (57–59) (Figure 3). The K63-linked ubiquitin chains on RIG-I promotes its interaction with “mitochondrial antiviral signaling protein” (MAVS, also known as CARDIF, IPS1, or VISA) and subsequent downstream activation of intracellular antiviral signaling (57, 59, 60) (Figure 3). K172 seems to play a central role in this process, since the K172R mutation severely reduces ubiquitination of the CARD domain of RIG-I and subsequent binding to MAVS (57, 59). Interestingly, phosphorylation of the CARD domains of RIG-I prevents CARD ubiquitination, indicating that phosphatases need to first dephosphorylate CARD-RIG-I and allow TRIM25-mediated polyubiquitination (61–64). In addition to anchored ubiquitin, RIG-I can be activated by binding through its CARD domains to unanchored ubiquitin chains assembled by TRIM25 (30). Following RIG-I activation, TRIM25-mediated K48-linked ubiquitination and subsequent proteasomal degradation of the larger MAVS isoform seems to be required for the downstream signaling that leads to type I IFN (65), although other studies have shown opposite results (66) (Figure 3). Studies in mouse embryonic fibroblasts deficient in TRIM25 demonstrated its importance in RIG-I activation and IFN-β production in response to viral infection (57). All those experiments demonstrated the essential role of TRIM25 in RIG-I activation and signaling. Nevertheless, the RIPLET E3 ligase has also been shown to be involved in RIG-I polyubiquitination and sustained activation (67, 68). RIPLET, such as TRIM25, has an N-terminal RING domain and a C-terminal SPRY domain. However, RIPLET lacks B-box domains and does not belong to the TRIM family. Although it has been reported that RIPLET can mediate K63 polyubiquitination of K172 in the CARD domain of RIG-I (67), it does not seem that TRIM25 and RIPLET play a redundant role in the activation of RIG-I, since RIPLET mainly generates K63 ubiquitin chains in the RIG-I C-terminal regulatory domain (68, 69). Some redundancy in RIG-I activation by different TRIM proteins may, however, exist. For example, TRIM4, which belong to the same subgroup as TRIM25 in the TRIM protein family and share with it similar structural characteristics, can also target RIG-I for K63 polyubiquitination in K154, K164, and K172 of its CARD domain, which results in the activation IRF3 and NF-κB, and IFN-β production (70).

TRIM25 may, however, have a dual role in RIG-I regulation, as it has been recently reported that TRIM25 negatively regulates RIG-I through stabilization of the ubiquitin-like protein FAT10. FAT10 non-covalently binds to RIG-I and sequesters it from the signaling platform inhibiting IRF3 and NF-κB activation (71) (Figure 3). This mechanism may contribute to limit the inflammatory response and reduce host damage, since FAT10 is accumulated subsequently to production of inflammatory cytokines late during virus infections (71).

TRIM25 also functions as an E3 ligase to conjugate the ubiquitin-like protein ISG15 to target proteins in a process termed ISGylation (72). Furthermore, auto-ISGylation of TRIM25 negatively regulates its ISG15 E3 ligase activity (73) (Figure 3). More than 300 proteins, both cellular and pathogen-encoded, have been identified as targets for ISG15 conjugation (74). In general, ISGylation, as well as free ISG15, has broad-spectrum antiviral effects (75–83), although a pro-viral effect has been described in the case of hepatitis C virus (84–86). Furthermore, human ISG15 has been shown to stabilize USP18, a negative regulator of the type I IFN receptor (87). In addition, it has been reported that some viruses have developed mechanisms to counteract the antiviral actions of ISG15, reflecting the importance of this molecule in the response against virus infections (88, 89).

In addition to RIG-I, TRIM25 also positively regulates the melanoma differentiation-associated protein 5 (MDA5)–MAVS–TRAF6 antiviral axis leading to activation of NF-κB (90). Also, it has been recently reported that the antiviral action of zing-finger antiviral protein (ZAP), a cellular protein that inhibits viral mRNAs translation, is enhanced by interaction with the SPRY domain of TRIM25 (91, 92).

TRIM25 may also associate with p53, and it has been proposed that this association promotes p53 degradation, since TRIM25 silencing increased the accumulation of p53 and reduced proliferation and migration of lung cancer cells (93). By contrast, other study showed that TRIM25 enhanced p53 levels by preventing their ubiquitination and proteasomal degradation (94). Despite increasing p53 levels, TRIM25 also inhibited p53 activity by preventing its acetylation by p300, an essential modification for the transcriptional activation of p53 target genes (Figure 3) (94). In any case, the regulation of p53 levels and/or activity by TRIM25 may have a deep impact on the innate immune response against infecting pathogens, as it has been reported that p53 upregulates the expression of interferon-stimulated genes (ISGs) either directly or through upregulation of IRF9, a component of the ISG factor 3 (ISGF3) (95–100).

An interesting observation is that TRIM25 can bind RNA through its central CC domain (101–103) (Figure 3). This opens new possibilities by which TRIM25 may influence intracellular signaling and/or replication of RNA viruses. It has been proposed that TRIM25 may use the RNA as a scaffold to get close to and modify its targets, including RIG-I and viral ribonucleoproteins (56). By contrast, as described below, some RNA viruses may take advantage of the capacity of TRIM25 to bind RNA to inhibit its function (104).

In conclusion, it is becoming evident that TRIM25 acts on multiple steps of signaling pathways inside the cell. Both positive and negative regulations of these pathways have been reported, adding complexity and relevance to the role of TRIM25 in the intracellular innate response. Pieces of evidence of the physiological relevance of TRIM25 *in vivo*, however, are indirect. For example, *TRIM25* polymorphisms have been associated with differences in the humoral response and secretion of some cytokines following measles virus vaccination in children (105); and, as described below, particular virus infections of mice also suggest a role of TRIM25 in regulating RIG-I *in vivo* (66, 106). Finally, the fact that TRIM25 has been under positive selection pressure in primates (107, 108) and that it is a targeted by some viral proteins (see next section) also indicates an important role of this molecule in the *in vivo* immunity against viruses.

## TRIM25 Activity is Controlled by Cellular and Viral Proteins

A strong innate immune response, including RIG-I-mediated signaling, is necessary for virus clearance but it has to be tightly regulated to prevent excessive inflammation. Therefore, it is not surprising that immune signaling pathways are regulated by numerous positive and negative interactions, many of which are targeted by virus proteins in order to facilitate virus replication.

Cyclophilin A (CypA), a peptidyl-prolyl *cis/trans* isomerase, positively regulates RIG-I signaling by two different mechanisms (66): (a) CypA promotes the interaction between RIG-I and TRIM25, which results in increased TRIM25-mediated K63-linked ubiquitination and activation of RIG-I (Figure 3) and (b) CypA competes with TRIM25 for the interaction with MAVS, which reduces TRIM25-induced K48-linked ubiquitination and proteasomal degradation of MAVS (Figure 3). Accordingly, infection of CypA knockout (KO) mice with Sendai virus resulted in reduced expression of type I IFNs and ISGs, higher viral load, and severer histopathology in lungs, as compared with wild-type infections (66). However, it remains to be elucidated whether these *in vivo* effects in KO mice are mediated by the lack of the CypA-dependent regulation of TRIM25 activity or by a different mechanism.

The linear ubiquitination assembly complex (LUBAC), composed of the E3 ligases heme-oxidized IRP2 ubiquitin ligase-1 (HOIL-1L) and HOIL-1-interacting protein (HOIP), negatively regulates RIG-I signaling using two different mechanisms (109): (a) LUBAC ubiquitinates the C-terminal SPRY domain of TRIM25, leading to TRIM25 degradation by the proteasome (Figure 3). TRIM25 ubiquitination depends on the RBR (RING-IBR-RING) domains of both HOIL-1L and HOIP; (b) The Npl4-type zinc finger (NZF) domain of HOIL-1L competes with TRIM25 for RIG-I interaction, thereby blocking ubiquitination and activation of RIG-I by TRIM25 (109) (Figure 3). Recently, the ubiquitin-specific protease 15 (USP15), a TRIM25-interacting protein, was reported to neutralize the inhibitory effect of LUBAC (110). USP15 binds to TRIM25 in viral infections, detaching the K48-linked ubiquitin chains assembled by LUBAC on TRIM25, thereby stabilizing the TRIM25 protein levels and promoting a sustained antiviral response (Figure 3).

TRK-fused gene (TFG) protein, which is another protein that interacts with TRIM25, has also been reported to inhibit the antiviral signaling mediated by RIG-I, although it is not sure that the interaction between TFG and TRIM25 itself is imperative for that effect (111).

It has been reported that some viral proteins are able to interact with TRIM25 and inhibit RIG-I activation. For example, the non-structural protein 1 (NS1) of influenza A virus (IAV) interacts with the CC domain of TRIM25 preventing its dimerization and K63-linked ubiquitination of the RIG-I CARDs, thereby suppressing RIG-I signal transduction (60, 106, 112) (Figure 3). This interaction seems to be mediated by residues E96/E97 in NS1, since an E96A/E97A NS1 mutant cannot interact with TRIM25, and also did not inhibit ubiquitination-mediated RIG-I signaling (106). Interestingly, in contrast to the wild-type virus, the E96A/E97A NS1 mutant was not virulent in mice (106). Since residues 96 and 97 have not been associated with other NS1 functions, it is tempting to speculate that the interaction between TRIM25 and NS1 is required for IAV virulence. In addition to its role in inhibiting RIG-I activation, the TRIM25-binding domain of IAV NS1 is needed for suppression of IL-1β secretion mediated by NLRP3 in macrophages, suggesting that TRIM25 may also be involved in the activation of this pathway (113, 114).

Another example of TRIM25 regulation by viral proteins is the N proteins of the severe acute respiratory syndrome coronavirus (SARS-CoV), which binds to the SPRY domain of TRIM25, thereby inhibiting activation of RIG-I by TRIM25 ubiquitination (115) (Figure 3).

Finally, it has been recently reported that subgenomic RNA from dengue virus binds TRIM25, preventing its USP15-dependent deubiquitination necessary for efficient RIG-I activation (104).

## Concluding Remarks

Emerging data show that the mechanisms by which TRIM25 may modulate the innate immune response against viruses are multiple and more complex than previously thought. TRIM25 has a dual role in RIG-I regulation: while TRIM25-mediated ubiquitination of RIG-I is essential for the transmission of downstream signaling, TRIM25 stabilization of FAT10 blocks active RIG-I. In addition, TRIM25 inhibits p53 acetylation, a modification indispensable for p53 antiviral activity. Finally, the ability of TRIM25 to bind RNA suggests new lines of investigation to uncover additional mechanisms of action and potential targets of this molecule. This complexity also increases the opportunities to develop novel strategies to regulate the innate immune response in order to reduce viral replication and/or avoid undesired excessive inflammation.

## Author Contributions

MM-V: investigation, resources and data curation, visualization, and writing-original draft preparation. SR: funding acquisition, writing-review, and editing. LM and AG-S: writing-review and editing. IM: conceptualization, funding acquisition, investigation, resources and data curation, project administration, supervision, visualization, writing-original draft preparation, writing-review, and editing.

## Conflict of Interest Statement

The authors declare that the research was conducted in the absence of any commercial or financial relationships that could be construed as a potential conflict of interest.
